# Evolving Management of Acute Pulmonary Embolism with Extracorporeal Membrane Oxygenation—A Narrative Review

**DOI:** 10.3390/jcm14228004

**Published:** 2025-11-11

**Authors:** Joseph P. Hart, Mark G. Davies

**Affiliations:** 1 Center for Quality, Effectiveness, and Outcomes in Cardiovascular Diseases, Houston, TX 77054, USA; mark.davies@ascension.org; 2 Division of Vascular and Endovascular Surgery, Medical College of Wisconsin, 8701 Watertown Plank Rd., Milwaukee, WI 53226, USA; 3 Department of Vascular and Endovascular Surgery, Ascension Health, Waco, TX 76710, USA

**Keywords:** ECMO, pulmonary embolism, management, outcomes

## Abstract

Acute pulmonary embolism (APE) carries significant 30-day mortality and morbidity. When APE is characterized by progressive hypoxia, hypotension, and right ventricular dysfunction, the risk of cardiovascular collapse and cardiac arrest is high, and intervention is recommended. As a result, there has been increasing impetus to utilize extracorporeal membrane oxygenation (ECMO) to provide rapid oxygenation support, immediate reduction in right ventricular (RV) overload, and hemodynamic support. Veno-arterial-ECMO modality is deployed to provide hemodynamic stability and restore tissue oxygenation and provides a bridge to recovery from percutaneous and open APE therapy. While many patients are placed on ECMO for a short period of time to treat APE, applying ECMO over an extended period pf time carries substantial multisystem morbidity due to systemic inflammatory response, hemorrhagic stroke, renal dysfunction, and bleeding. It appears that the initiation of ECMO alone, with or without administration of systemic thrombolysis, will not improve outcomes over conventional therapy for high-risk APE. The current literature demonstrates that ECMO is best paired with open or percutaneous thrombectomy to reduce or eliminate the clot burden and rapidly stabilize cardiovascular status; these dual outcomes translate into patient survival. However, a series of meta-analyses have not demonstrated that the use of ECMO in hemodynamically unstable APE results in a significant survival advantage compared to patients treated without ECMO.

## 1. Introduction

Pulmonary embolism (PE) remains a common disease with a yearly incidence rate ranging from 39 to 115 per 100,000 population [1,2], and it remains a leading cause of cardiovascular mortality in the United States with 300,000 deaths per year [1,3]. The predominant focus of health systems in recent times has been the development and implementation of prophylactic DVT pathways to mitigate the development of an APE. Hemodynamically stable APE has an associated 30-day mortality of 1% to 2%, while hemodynamically unstable APE is associated with a 30-day mortality of 10% to 25% [4]. There is an associated 30-day mortality rate ranging from 16 to 46% for patients in shock, approaching 52 to 84% for those with cardiac arrest [5,6,7,8,9,10,11,12,13]; Multivariable regression analysis demonstrated vasopressor use, extracorporeal membrane oxygenation use, identified clot-in-transit, and malignancy as factors associated with in-hospital mortality [14]. Most recently, the PERT consortium registry reported that pulmonary embolism in participating centers had a distribution as follows: 19% low risk, 34% low-intermediate risk, 35% intermediate-high risk, and 12% high risk. The proportion of patients who were offered advanced therapy ranged from 16% to 46%, and the 30-day mortality was 16%, with a range of 9% to 44% [15]. The need to control hemodynamic instability in APE has prompted the increasing use of Extra-Corporal Membrane Oxygenation (ECMO) as a means of immediate cardiopulmonary support [16,17]. There has been a substantial increase in the literature on ECMO for APE over the last decade, which has prompted this narrative review to provide an update on the current state of ECMO in the management of APE. This narrative addresses the role of veno-arterial ECMO in APE using the PICO (Population, Intervention, Comparison, Outcome) framework and poses the following question. In patients with massive pulmonary embolism and hemodynamic collapse (P), does early use of VA-ECMO as part of a reperfusion strategy (I), compared to standard advanced critical care support coupled with reperfusion therapy, (C) improve survival (O)?

## 2. Bibliographic Strategy

To develop this narrative review, a bibliographic search of OVID© MEDLINE©, Scopus©, and Cochrane© databases was performed using the search terms “Extra-Corporal Membrane Oxygenation”, “ECMO”, “Pulmonary Embolism”, and “Venothromboembolism” to identify all publications in the English language between January 2010 to June 2025 following the Preferred Reporting Items for Systematic Reviews and Meta-Analyses guidelines [18].

## 3. Classification

APE can be classified by size or mortality risk. The initial American Heart Association (AHA) classification introduced a three-level classification, Minor, Submissive, and Major APE, based on their presenting anatomic and physiological findings [19]. The greater the clot burden, the worse the hemodynamic instability. More recently, a classification based on the risk of mortality was introduced by the European Society of Cardiology (ESC): low-risk, intermediate-risk, and high-risk APE. Risk stratification of patients with APE requires the assessment of clinical presentation, imaging of the chest and right ventricle, and biomarkers of myocardial stress (troponin and B-type natriuretic peptide) and end-organ perfusion (serum lactate) in the context of a patient’s comorbidity burden and other aggravating conditions that can adversely affect early prognosis [4]. The BOVA Score is a tool used to assess the risk of complications and mortality in patients with APE who are hemodynamically stable [20]. It categorizes patients into low-, intermediate-, and high-risk groups based on four specific clinical criteria: heart rate, systolic blood pressure, cardiac troponin, and right ventricular dysfunction [21]. The BOVA score has been demonstrated to effectively discriminate normotensive APE with different short-term prognoses and can identify patients at higher risk of short-term adverse events [22]. However, there are no papers that state that the BOVA score is valuable in managing patients with unstable APE. It is always important to define APE as hemodynamically stable or unstable. Currently, hemodynamic instability is defined as a systolic blood pressure of less than 90 mmHg, hypotension requiring vasopressor support, or a decrease in systolic blood pressure of more than 40 mmHg for more than 15 min, the requirement for inotropic support, or persistent profound bradycardia [4,19,23]. Presentation of a high-risk APE is characterized by cardiac arrest, obstructive shock with end-organ hypoperfusion, or persistent hypotension, and it is these patients who are the focus of ECMO therapy. Within the ESC guidelines, there is a recommendation to evaluate the right ventricle (RV) even in patients with a low Pulmonary Embolism Severity Index (PESI) score or a simplified Pulmonary Embolism Severity Index (sPESI) of 0 [4].

## 4. Pathophysiology

Sixty-three percent of patients with APE present with severe hypoxemia [24,25]. An APE leads to an increase in pulmonary artery resistance, interferes with pulmonary gas exchange, and results in changes in lung mechanical capacity [16,24,26,27]. Once lodged in the vascular bed, the embolic material creates a mechanical obstruction, induces hypoxic and acidotic-induced vasoconstriction, and triggers the release of vasoactive mediators from pulmonary artery endothelial and smooth muscle cells. The acute and rapid increase in pulmonary vascular resistance drives acute RV pressure overload and subsequent RV failure [28]. The dysfunctional and dilated RV impacts left ventricular (LV) filling and significantly decreases LV preload, manifesting in decreased cardiac output, systemic hypotension, and, ultimately, cardiogenic shock. The goal of ECMO in the setting of hemodynamic instability induced by APE is to reduce RV pressure, restore normal LV function, and improve systemic hypoxia. This will restore cardiac output, improve blood pressure, and reverse the characteristics of cardiogenic shock and end-organ malperfusion.

## 5. Evidence-Based Guidelines and Risk Stratification for ECMO in APE

The primary indication for ECMO in the management of APE is the treatment of cardiogenic shock and witnessed cardiac arrest. Numerous contraindications have been established for the use of ECMO in these circumstances and are shown in Table 1 and Table 2 [29,30]. Evidence-based guidelines for the therapy of APE have been developed by the American College of Chest Physicians (ACCP), American Heart Association (AHA), and the European Society of Cardiology (ESC) [31,32,33]. When dealing with hemodynamically significant APE in the intermediate and high risk categories, consensus and evidence recommend intervention with systemic thrombolysis [34], catheter-based techniques [35] or surgical embolectomy [36]; ECMO is recommended for cardio-pulmonary support to facilitate these interventions in patients who are suitable candidates for ECMO [37].

Once there is a clear indication for ECMO in APE, the patient should be risk-stratified for the potential of mortality and morbidity using available predictive algorithms. Currently, there are two predictive scoring tools suitable for the hemodynamically unstable APE patient being considered for ECMO: Survival after VA-ECMO (SAVE) and Sequential Organ Failure Assessment-Right Ventricle (SOFA_RV_). The current SAVE score incorporates the patient’s age, body weight, etiology of cardiogenic shock, presenting neurological function, renal function, presence of metabolic acidosis, respiratory and cardiac parameters, presence of end-organ failure, and serum lactate to classify patients into five mortality categories, ranging from 18% to 75% (Table 3) [38]. A SAVE score of ≤−10 is associated with a mortality of less than 20%, while a SAVE score > 5 is associated with a 75% mortality. The current SOFA ECMO score (SOFA-right ventricle (RV)—SOFA_RV_) incorporates the current SOFA components and an echocardiographic assessment of the right ventricle (Table 4) [39]. SOFA_RV_ outperformed the original SAVE without a lactate in predicting mortality in patients on VA-ECMO. A SOFA_RV_ score < 5 is associated with a mortality of less than 20%, while a score > 14 is associated with a mortality of 95% (Table 3). In the setting for ECMO and APE, neither the PESI nor the sPESI has been shown to be helpful in clinical decision-making in the APE patient placed on ECMO.

## 6. Goals of and Implementation Strategies for ECMO Care in APE

**Figure 1:**
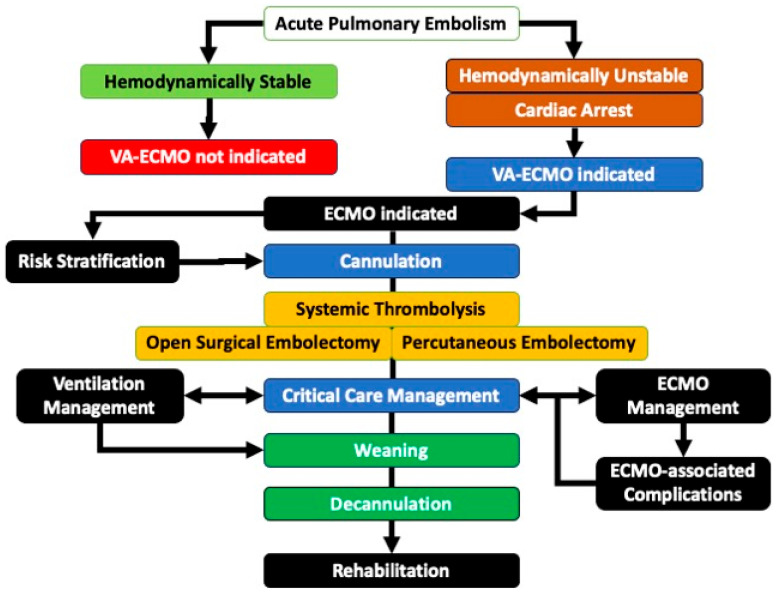
Algorithm for management of ECMO in Acute Pulmonary Embolism. In hemodynamically Acute Pulmonary Embolism, ECMO is not indicated. In hemodynamically unstable patients or those presenting with cardiac arrest, VA-ECMO is indicated, provided there are no contraindications. Patients should be risk-stratified, and the best practice cannulation techniques should be employed to ensure safe and effective access. Once the patient is stable on VA-ECMO, APE therapy can be performed. The patient is then managed in the intensive care unit, which includes ventilation management and ECMO-associated complications. Patients who survive and recover are weaned and then decannulated before transferring to an inpatient unit. Most will need care in an acute rehabilitation unit.

The goals of ECMO in hemodynamically unstable APE are to restore circulation, reduce the right ventricle afterload, and restore end-organ perfusion and oxygenation. VA-ECMO will rapidly decrease RV overload (leading to restored RV function), increase tissue perfusion, and enhance overall oxygenation. In the majority of cases with hemodynamically unstable APE, the cardiovascular status of the patient improves significantly within the first 24 h on ECMO, even if no reperfusion intervention is implemented [40]. There are three potential implementation strategies for ECMO in the hemodynamically unstable APE: bridge to definitive therapy, sole therapy, and recovery after treatment. The first and most common strategy provides pre-operative and peri-operative support for either percutaneous thrombectomy or open surgical embolectomy. The second strategy, which is implemented least often, supports the patient while they receive anticoagulation or systemic thrombolysis without the need for open or percutaneous intervention. The third strategy is to support the patient after intervention, allowing for RV recovery, restoration of pulmonary function, and end-organ recovery once the thrombus burden has been removed by pharmaceutical, percutaneous or open modalities. An additional scenario is becoming more common in practice: the pre-emptive strategy has been described as involving the mobilization of the ECMO team and equipment, with placement of ECMO cannulae for VA-ECMO prior to percutaneous intervention to rapidly support hemodynamic collapse during percutaneous thrombectomy for hemodynamically unstable APE. A proposed algorithm for VA-ECMO care in APE is shown in Figure 1.

## 7. Complications of ECMO

All patients on ECMO can experience a cascade of complications that may affect their potential for survival and often require substantial additional resources to manage these issues as they arise [41]. The longer the patient is on ECMO, the greater the risk for the development of complications. Most patients in ECMO for APE will have short ECMO runs but can still experience common patient-related complications. These common patient-related complications include the need for blood and blood product transfusions ranging from 47% to 100%, the occurrence of acute limb ischemia ranging from 10% to 70%, and significant neurologic events of variable and escalating severity ranging from 6% to 18% (Table 4) [42]. The longer the need for ECMO support, the greater the risk of the development of multi-organ system failure, which is reported to range from 30% to 80%. Common life-threatening mechanical complications can occur in 4.0% of ECMO cases. These reports include accidental decannulation (1.3%), abrupt falling circuit flows (1.1%), pump failure (1.1%), circuit rupture (0.4%), and air in the circuit (0.2%) (Table 4) [43]. Most (90.9%) of these device issues required circuit and cannula change, while 9.1% required vein collapse relief procedures (adjusting the position of the cannulae or using additional cannulation to improve blood flow). When all factors are analyzed, awake ECMO and long-term ECMO support were found to be significantly associated with these life-threatening mechanical complications [43].

## 8. Management

Management of ECMO patients is performed in an intensive care environment under the supervision of a multidisciplinary team. The duration of VA-ECMO support for APE patients has been reported to be in the range of 3 to 7 days. Some patients may be weaned within 24–48 h, while others may require support for longer periods. The principal areas of management are circulatory and ventilatory support. This involves managing the ECMO system, supplementing circulatory support with fluids and/or vasoactive agents, and providing oxygenation support with protective ventilatory practices. APE is characterized by hypoxemia, and high-flow oxygen therapy or noninvasive mechanical ventilation are the preferred methods, which can usually provide adequate oxygenation to a target oxygen saturation of >90%. In the recently published Air vs. Oxygen for Intermediate Risk Pulmonary Embolism (AIR) trial, fixed-dose supplemental oxygen was compared with ambient air in normoxic patients with APE but with RV dysfunction on echocardiography; there was a nonsignificant trend toward improvement in the RV to LV ratio—a measure of RV dysfunction—with supplemental oxygen. However, many patients will require intubation due to their initial presenation with cardiac arrest or increasingly severe hemodynamic instability, and those patients with a failing RV are prone to peri-intubation hemodynamic collapse. Once intubation and mechanical ventilation are initiated, tidal volumes should be maintained at 6 to 8 mL/kg ideal body weight to avoid overdistention, and positive end-expiratory pressure (PEEP) should be applied cautiously because PEEP reduces RV preload, exacerbating RV dysfunction. Hemodynamic support is best achieved with norepinephrine and supplemental vasopressin, with intravenous fluids used sparingly. The addition of inhaled nitric oxide NO can be considered in patients with ongoing RV dysfunction despite hemodynamic support, who have documented euvolemia, and are receiving supplemental oxygen administration. Ongoing management of hematologic and metabolic factors, end-organ dysfunction, and nutrition occurs during and after ECMO. Additional consultation and management of the numerous device-associated and patient-related complications associated with ECMO are also necessary as the patient recovers from the primary insult of the APE. Integrated into this complex management is communication with the patient and family, linked to the utilization of social work, medical ethics, palliative care, and pastoral services.

## 9. Weaning and Decannulation

Weaning from ECMO is the successful cessation of the mechanical support and decannulation of the patient. Decisions to wean from VA-ECMO are as critically important as the institution of ECMO [37]. Any consideration of a weaning trial should not be attempted within the first 48 h and only when the patient has sufficiently recovered from their underlying etiology and demonstrated evidence of sufficient cardiopulmonary recovery [44]. In the case of APE, the dominant variable is related to recovery of right ventricular function and reversal of end-organ malperfusion. The criteria to wean from VA-ECMO are shown in Table 5. Weaning is typically a gradual process, and adjustments are made based on the patient’s tolerance and ability to maintain stable respiratory and hemodynamic status [45]. Factors associated with a successful or failed weaning process are shown in Table 6 [46,47].

A weaning test from VA-ECMO decreases the ECMO flow rate to 2 L/min for at least 60 min. If the mean arterial pressure drops more than 10–15 mmHg or falls below 65 mmHg, the test is considered a failure [48]. During the weaning process, the FiO_2_ on the ECMO circuit is decreased, the sweep gas flow through the oxygenator is also reduced, and the patient is transitioned to a conventional mode on the ventilator, adjusting tidal volume, respiratory rate, PEEP, and introducing pressure support/pressure control to provide sufficient ventilatory support [49]. Successful weaning is defined as a patient with no further need for VA-ECMO, cardiopulmonary transplantation, or placement of an LVAD at 30 days. In the literature, weaning rates range between 35% and 70%. It is important to note that 20% to 65% of patients weaned from VA-ECMO for all causes including APE do not survive until hospital discharge due to insufficient myocardial recovery, primary or secondary multiorgan failure, neurological damage, and other comorbidities [50,51,52,53,54]. A history of diabetes, previous myocardial infarction, prolonged ECMO support, and hypoxemia at ECMO weaning are noted to be independent predictors of in-hospital mortality after weaning [55].

Decannulation should promptly occur after a successful weaning trial to avoid potential complications associated with ECMO support. A decannulation test from VA-ECMO is performed after the patient has been successfully weaned and tolerated a trial of ECMO at 2 L/min for 8 h or greater with sustained and stable hemodynamic and ventilatory function. Circuit flow is gradually decreased to 1 L/min for approximately 1 min to detect hemodynamic instability with minimal ECMO support. If this reduction is tolerated, blood flow is returned to 2 L/min, and decannulation is scheduled. Currently, removal of cannulae for VA-ECMO can be achieved by either percutaneous closure devices or open surgical repair of the vessels. Rates of vascular complications at the time of decannulation range between 8% and 20% [56]. Following ECMO decannulation, many patients experience a renewed systemic inflammatory response syndrome, defined as having any 2 out of the 3 of these findings after ECMO decannulation (without regard to the presence of infection): fever (temperature > 101.5 °F), leukocytosis (white blood cell {WBC} > 12,000, or 25% increase from pre-decannulation baseline), and escalation of vasopressors compared to the patient’s pre-decannulation baseline. Significant complications at the cannulation site remain a major issue during and after decannulation for VA-ECMO [57]. Rates of vascular complications at the time of decannulation range between 8% and 20% [56]. The risk factors linked to the occurrence of a vascular complication following percutaneous closure are female gender, the presence of peripheral arterial disease, and duration of ECMO [58].

## 10. Outcomes

In a study of the U.S. Nationwide Inpatient Sample (NIS) covering the years 2016–2020, involving 122,735 hospitalizations, high-risk APE hospitalizations were identified. Among these, ECMO was used in 2.3% of cases, with ECMO as a stand-alone therapy in 1.4%, thrombolysis-based reperfusion in 0.4%, and mechanical reperfusion in 0.5%. The use of ECMO was associated with a reduction in in-hospital mortality. When compared with reperfusion intervention alone or placement on ECMO alone, ECMO plus thrombolysis-based reperfusion was associated with reduced in-hospital mortality. However, no difference was found between ECMO plus mechanical reperfusion and ECMO as a stand-alone treatment, and both were linked to increased in-hospital mortality [59].

In the literature of ECMO performed in adults with APE, most reports consist of single-institution experiences or registry data [60]. The heterogeneity in the literature regarding indications for ECMO, patient populations enrolled, ECMO techniques utilized, and the lack of randomized clinical trials leaves the fundamental question of which adult populations with APE may benefit from ECMO largely unanswered [61]. Furthermore, most current reports on the use of ECMO in adults suffer from small sample sizes, retrospective designs, and the absence of a historical or control cohort [61]. The strongest data on ECMO in APE comes from meta-analyses of the current literature (Table 7).

In 2015, Yusuff and Associates [62] reported a systematic review on ECMO and found an overall survival of 70.1%. Survival with ECMO was equivalent, irrespective of the adjuvant intervention used to remove pulmonary clots: thrombolysis, catheter-based embolectomy, or surgical embolectomy. Those who had ECMO initiated while in cardiac arrest, however, had an overall higher mortality compared to all other patients. In 2020, Baldetti conducted a pooled analysis of all published experiences of ECMO support in hemodynamically unstable APE (21 studies with 635 patients) and showed that immediate ECMO support was pursued in 61.9% of patients with cardiac arrest, and 57.0% of these patients underwent adjunctive reperfusion therapies. Early all-cause mortality in hemodynamically unstable APE was 41.1%, and in meta-regression analyses, no covariates were associated with mortality. In 2021, Harwood Scott et al. reported on 301 patients experiencing APE-related cardiac arrest [66] and found that only 61% of the patients survived to discharge. Patients who received systemic thrombolysis for MPE before ECMO cannulation had similar survival rates compared with those who underwent ECMO cannulation without exposure to systemic thrombolysis. The authors also noted that there was no significant difference in risk of death if ECMO cannulation occurred in the emergency department or other hospital locations. In an associated multivariate analysis, the authors demonstrated a 3-fold increase in the risk of death for patients over 65 years old and a 6-fold increase if cannulation occurred during cardiopulmonary resuscitation. In 2022, Kaso et al. [68] reported a meta-analysis of in-hospital mortality in patients treated for hemodynamically unstable acute pulmonary embolism (APE) who received either treatment with or without extracorporeal membrane oxygenation (ECMO). Eleven eligible studies with 791 patients were included (270 subjects received ECMO, and 521 subjects did not; 64% presented with a cardiac arrest). In-hospital mortality was found not to be significantly different between patients treated with and without ECMO. In a 2022 systematic review of the success of reperfusion strategies in APE in combination with ECMO, 17 studies with 327 patients met the inclusion criteria. A third of patients underwent mechanical pulmonary reperfusion, of which the majority were an open surgical embolectomy, while two-thirds underwent percutaneous thrombectomy and/or systemic thrombolysis. The overall mortality rate was 22.6% in the mechanical reperfusion group and 42.8% in the percutaneous thrombectomy and/or systemic thrombolysis group [69]. These findings were extended by Boey and colleagues [70], who conducted a systematic review and meta-analysis of 39 observational studies with a study population of 6409 patients receiving VA-ECMO for high-risk APE. The pooled mortality of this cohort was 42.8%. Patients treated with ECMO and catheter-directed therapy (28.6%) had significantly lower mortality (*p* < 0.0001) compared to those treated with ECMO and systemic thrombolysis (57.0%). Cardiac arrest prior to ECMO initiation (*p* = 0.018) and pre-ECMO heart rate (*p* = 0.0003) were significantly associated with mortality. The pooled risk ratio when comparing mortality between patients on ECMO and those not on ECMO was 1.51-fold in favor of ECMO. The pooled mortality rate was 55.2%, as determined by trim-and-fill analysis, which accounted for the significant publication bias. It appears that the initiation of ECMO alone, with or without administration of systemic thrombolysis, will not improve outcomes over conventional therapy for high-risk APE. As a result, it appears that ECMO is best paired with an open or percutaneous thrombectomy to reduce or eliminate the clot burden and rapidly stabilize cardiovascular status, which is linked to survival.

Readmission rates and predictors of readmissions in patients presenting with cardiogenic shock who required VA-ECMO are sparse, and there is no firm data on those who required VA-ECMO for hemodynamically unstable APE. In a study of the United States Nationwide Readmission Database (2016–2018) of patients who had been placed on ECMO, the readmission rate was 23.9% with a median time from discharge to readmission of ten days [72,73]. There were multiple medical issues that led to readmission: cardiovascular causes (31.6%), complications of medical or device care (17.7%), presence of infection (11.3%), or gastrointestinal/liver complications (10.1%) [72]. Survival rates at 30 days, 1 year, and 3 years after weaning from VA-ECMO placed for all causes have been reported at 59%, 46%, and 41%, respectively [74].

There is limited data on the long-term outcomes of APE after ECMO. Stadlbauer et al. reported on 119 high-risk APE patients supported by VA-ECMO (67% had ECMO during or after cardiopulmonary resuscitation) [75]. The overall survival rate was 45.4%. At a median follow-up of 54 months, echocardiography did not reveal any signs of RV dysfunction or pulmonary hypertension, with only slightly decreased pulmonary function testing. Seventy-four percent of patients showed no limitations in motor activity and mobility; however, they did exhibit slightly impaired quality of life scores compared to an age-matched control population. Corsi et al. reported similar findings in seven patients at a mean follow-up of 19 months [76].

## 11. Conclusions

Provision of ECMO in hemodynamically unstable APE is now considered an important part of an interventional APE program, where it is deployed to stabilize a patient prior to intervention or rescue a patient after intervention. When considering placing a patient on ECMO, risk stratification is important and should be performed in the context that is associated with substantial multisystem morbidity and resource utilization. To date, there have been no significant differences in outcomes between patients treated with and without ECMO in meta-analyses. While ECMO for hemodynamically unstable APE now has a place in current guidelines, it should be combined with an aggressive PE interventional program and strictly adhere to the risk stratification and selection criteria for ECMO to achieve optimal results.

## Figures and Tables

**Table 1:** Absolute and relative contraindications to all forms of ECMO.

Absolute	Relative
Patient refusal	Advanced age
Advanced stage of cancer	Immunosuppressed patients or on pharmacological immunosuppression
Non-survivable intra-cerebral hemorrhage Non-survivable cerebral herniation Intractable intracranial hypertension	Injurious ventilator settings > 7 days
Irreversible destruction of the lung parenchyma	Right-heart failure
Contraindications to lung transplantation	Hematologic malignancies
	SAPS II score ≥ 60 points
	SOFA score > 12 points
	PRESERVE score ≥ 5 points
	RESP score ≤ −2 points
	PRESET score ≥ 6 points
	Active do not attempt resuscitation status

SAPS II, Simplified Acute Physiology Score II; SOFA, Sequential Organ Failure Assessment; PRESERVE, Predicting death for Severe ARDS on VV-ECMO; RESP, Respiratory ECMO Survival Prediction; PRESET, Prediction of Survival on ECMO Therapy.

**Table 2:** Contraindications to ECMO in APE-induced cardiogenic shock and cardiac arrest.

Cardiogenic Shock	Cardiac Arrest
Aortic valve incompetence	Older than 70 years old
End-stage heart failure	Unwitnessed arrest
Severe chronic obstructive pulmonary disease (COPD)	An interval from cardiopulmonary arrest to first CPR of greater than 5 min
End-stage liver failure	Observed initial rhythm does not conform to Ventricular Fibrillation (VF), paroxysmal ventricular tachycardia, or pulseless electrical activity (PEA)
End-stage renal failure (ESRD)	Presence of recurrent VF
Terminal or irreversible illness	Intermittent return of spontaneous circulation

**Table 3:** ECMO and SOFA _RV_ predicted mortality.

Modified SAVE Score	Predicted Mortality	SOFA_RV_ Score	Predicted Mortality
>5	25%	0–1	0%
1 to 5	43%	2–3	6%
−4 to 0	58%	4–5	20%
−9 to −5	70%	6–7	22%
≤−10	72%	8–9	33%

SAVE, Survival after Veno-Arterial ECMO; SOFA_RV_, Sequential Organ Failure Assessment.

**Table 4:** Complications of ECMO therapy.

Patient		
System	Mechanisms	Complication
Inflammatory	Activated coagulation cascade, Activated complement systems, Activated endothelial cells, activated leukocytes, Activated platelets	Microcirculatory thrombosis Microcirculatory dysfunction Aseptic parenchymal inflammation
Coagulation	Thrombocytopenia, Platelet dysfunction, Acquired von Willebrand syndrome, Hemolysis, Enhanced fibrinolysis	Bleeding Thrombosis End-organ malperfusion
Gastrointestinal Tract	Dysfunctional gastrointestinal barrier	Bacterial translocation and endotoxin release
Renal	Hypotension, Hypoxia, Ischemia–reperfusion injury, Parenchymal ischemia	Acute kidney injury Dialysis
Pulmonary	LV overload, Systemic inflammatory response, Hemodynamic changes parenchymal ischemia, lung congestion due to altered ventricular filling, ischemia–reperfusion injury	Pulmonary injury, pulmonary congestion
Limb	Mechanical obstruction, In situ thrombosis, Distal embolism	Acute arterial ischemia Acute Compartment syndrome Acute DVT Phlegmasia Thrombo-embolism
Neurologic	Hypotension, Hypoxia, Bleeding, Thrombosis, Parenchymal ischemia, Ischemia–reperfusion injury	Intracranial hemorrhage Acute ischemic stroke New-onset seizure activity Cerebral edema Intracranial hypertension Hypoxic–ischemic encephalopathy
Device related		
Circuit:	Incorrect connections; clamped tubing	Poor Flow Blood Loss Air embolism Hypoxia
Oxygenator	Perforations or leaks, Blood cell trauma	Air embolism Hemolysis
Sweep Gas Flow	Flow too high or too low	Abnormal gas exchange
Pump	Leak, Malfunction	Hemorrhage Thrombosis Poor Flow Hypoxia
Cannulation related	Vessel injury, Vessel obstruction	Thrombus Ischemia Macro- and micro-embolism
Cannulas	Misplacement, Size mismatch, Displacement	Reduced Flow Thrombus Bleeding
Heat Exchanger	Heating/cooling malfunction	Hypothermia/hyperthermia
Monitoring Devices	Malfunction	Poor Flow Hypoxia

**Table 5:** Criteria to wean from ECMO.

Hemodynamic Stability	Present
Vasoactive, inotropic support	Minimal
Pulse pressure	>10 mm Hg
Mean arterial pressure	>65 mm Hg
Echocardiographic imaging	Satisfactory RV and LV function
PaO_2_ with an FiO_2_ of 21%	>60 mmHg
ECMO flow	3–4 L per min
Sweep gas flow	1 L per min
Acute respiratory distress syndrome	No clinical or radiological signs

**Table 6:** Parameters impacting weaning.

**Parameters are Associated with Successful Weaning**	
Aortic velocity time interval (VTI)	≥10 cm
Left ventricular ejection fraction	>25%
Lateral mitral annulus peak systolic velocity	>6 cm/s
**Parameters are associated with failure of weaning**	
Pre-existing ischemic heart disease	Present
Pre-wean test left ventricular ejection fraction	≤25%
Post-wean test left ventricular ejection fraction	≤40%
Post-wean test systolic blood pressure	≤120 mmHg
Duration of ECMO support	>7 days

**Table 7:** Systematic reviews and meta-analysis on the role of ECMO in APE.

Author	Year	Reference	Study Type	Number of Studies	Number of Patients	ECMO	No ECMO	Mean ECMO Duration (Days)	Complication	Successful Wean	ECMO Survival	No ECMO Survival
Yusuff	2015	[62]	SR	19	69	69	none	5	23%	NR	70%	none
Baldetti	2020	[63]	SR/MA	21	416	416	none	2	25%	69%	59%	none
O’Malley	2020	[64]	SR	50	99	99	none	3	23%	NR	88%	none
Pozzi	2020	[65]	SR/MA	16	533	533	none	3	19%	68%	50%	none
Harwood Scott	2021	[66]	SR	77	301	301	none	NR	7%	85%	61%	none
Karami	2021	[67]	MA	20	1947	1138	809	NR	NR	NR	57%	65%
Kaso	2022	[68]	MA	11	791	270	521	4	NR	NR	54%	60%
Chopard	2022	[69]	SR/MA	17	327	106	221	NR	22%	NR	77%	57%
Boey	2023	[70]	SR/MA	39	1,177,998	6409	1,171,589	4	51%	NR	45%	81%
Yang	2025	[71]	MA	10	2846	1181	1685	NR	NR	NR	67%	65%

SR, Systemic Review; MA, Meta-analysis; NR, Not reported.

## Abbreviations

The following abbreviations are used in this manuscript:

APE	acute pulmonary embolism
ECMO	extra-corporal membrane oxygenation
VA-ECMO	veno-arterial extra-corporal membrane oxygenation
